# Outcomes of reducing stigma towards alcohol misuse during adolescence: results of a randomized controlled trial of the MAKINGtheLINK intervention

**DOI:** 10.1186/s13034-020-00317-7

**Published:** 2020-03-21

**Authors:** Ali Cheetham, Emma Sandral, Dan I. Lubman

**Affiliations:** 1 Turning Point, Eastern Health, 110 Church St, Richmond VIC, Richmond, VIC 3131 Australia; 2 Monash Addiction Research Centre and Eastern Health Clinical School, Monash University, Box Hill, VIC Australia

**Keywords:** Adolescence, Intervention, Stigma, Alcohol, Help-seeking

## Abstract

**Background:**

While it is well-recognized that the stigma associated with alcohol use problems can prevent or delay help-seeking, there is limited research examining stigmatising attitudes towards alcohol misuse, or their consequences, during adolescence. The current study examined the results of a school-based intervention on adolescents’ stigmatising attitudes towards alcohol misuse among their peers, and how changes in attitudes influenced intentions to encourage help-seeking, as well as participants’ personal use and misuse of alcohol.

**Methods:**

Participants (n = 463) were a subset of a larger sample participating in a randomized controlled trial of the MAKINGtheLINK intervention. Of the included participants, 287 (62%) were allocated to the intervention group and 176 (38%) to the control group. Assessments were conducted at baseline and 6-weeks, 6-months, and 12-months post-baseline. At each assessment, participants were presented with a vignette describing a peer experiencing alcohol misuse and completed the General Help Seeking Questionnaire as well as a 10-item scale measuring stigmatising attitudes. Alcohol use was also assessed.

**Results:**

The intervention was associated with a greater reduction in ‘weak-not-sick’ attitudes over time, which in turn predicted stronger intentions to encourage help-seeking from family members and formal help sources at the 12-month follow-up. Perceptions of dangerousness did not change significantly as a result of the intervention, however overall perceptions of dangerousness demonstrated a trend towards encouraging help-seeking from formal sources. Changes in stigma were not associated with past-year alcohol use or problems.

**Conclusions:**

School-based interventions such as MAKINGtheLINK can decrease some stigmatising attitudes towards alcohol misuse during adolescence, and increase adolescents’ intentions to encourage help-seeking from both formal and informal help sources. However, results varied depending on both the dimension of stigma examined and the type of help source, highlighting a complex relationship between stigma, intentions, and sources of help that requires further investigation. Importantly, reducing stigma did not appear to result in negative effects due to greater acceptance of drinking (e.g., heavier alcohol use), supporting continued efforts to reduce alcohol-related stigma during adolescence.

*Trial registration*: Registered with the Australia and New Zealand Clinical Trials Register (ANZCTR) on the 27th of February 2013 (ACTRN12613000235707)

## Background

There is considerable evidence that alcohol use disorders are more severely stigmatized than other forms of mental illness. Compared to depression and psychosis, individuals experiencing alcohol use disorders are perceived to be more dangerous and unpredictable, and are more likely to be considered ‘weak’ as opposed to ‘sick’ and in need of treatment [1, 2]. Research from the mental health literature has highlighted the adverse outcomes of stigmatising attitudes and beliefs, which can include discrimination, social isolation, delayed problem recognition, and avoidance of professional treatment [3, 4]. Together, this literature highlights a need to reduce the stigma associated with alcohol misuse.

In particular, there is a need to understand and address alcohol-related stigma in young people. While a number of interventions aiming to reduce the stigma associated with substance misuse have been trialed in adult populations (see Livingstone et al. [5] for a review), there has been limited research examining stigmatising attitudes towards alcohol misuse among adolescents. However, the existing evidence appears to be consistent with the adult literature [6, 7]. For example, adolescents report a preference to avoid alcohol-misusing peers, as well as weaker intentions to encourage them to seek help. As the negative attitudes that have been formed in adolescence often persist into adulthood [8], it is important to counter alcohol-related stigma as early as possible. Moreover, as young people often rely on peers rather than seeking professional help [9], it is vital that they are not ostracized or provided with unhelpful advice due to the stigma associated with alcohol misuse.

Schools are excellent sites for health promotion activities, as they provide an opportunistic setting in which to reach out to large groups of young people [10]. As alcohol misuse increases markedly around the age of 15–16 years [11], interventions conducted at this time point have the potential to address stigma at a crucial developmental period, in which adolescents are beginning to form attitudes towards alcohol misuse and related problems [12]. Teaching young people how to support their peers during this period may help reduce the stigma that has been associated with alcohol misuse and its consequences, as well as encouraging help-seeking from appropriate sources (i.e., health professionals, GPs).

Recently, we reported on the outcomes of a cluster randomised controlled trial of the MAKINGtheLINK intervention, a school-based program that teaches adolescents how to overcome barriers to accessing help for mental health and substance use problems and encourages professional help-seeking [13]. At 12-months post-baseline, intervention participants were significantly more likely to have sought help from formal sources (i.e., from trained professionals) than informal sources (i.e., friends or family), suggesting that MAKINGtheLINK improves the quality of help-seeking among adolescents experiencing mental health problems. The intervention was also associated with increased confidence in helping a friend up to 6 months post-baseline [14]. Two dimensions of stigma were examined as part of the intervention: ‘weak-not-sick’ (i.e., that the problems experienced are a sign of personal weakness) and perceptions of dangerousness (i.e., that the problems experienced mean that individual is dangerous and unpredictable). In analysis of baseline data from the same sample, we found that ‘weak-not-sick’ attitudes were associated with weaker intentions to encourage help-seeking for alcohol misuse from peers, family members, formal (professional) help sources, and the internet, while ‘dangerousness’ attitudes were associated with weaker intentions to encourage help-seeking from peers and the internet, but stronger intentions to encourage help-seeking from formal sources [15]. These findings are in line with past research examining the relationship between stigma and help-seeking attitudes and behaviors during adolescence [16, 17].

Potentially, reducing alcohol-related stigma during adolescence could increase young people’s confidence and willingness to help their peers. However, it is important to note that doing so may also produce negative effects, such as increasing harmful use and associated problems due to greater acceptance of drinking. For example, Adlof and colleagues found that stigma declines amongst adolescents with drug-using friends, particularly during mid-adolescence (i.e., 15–16), while stigma against individuals with substance use disorders is inversely related to the prevalence of substance use amongst close friends [18]. As noted, negative attitudes towards substance users may be difficult to maintain if peers that adolescents’ have close relationships with, or are heavily influenced by, begin using alcohol or other drugs. This alone may increase willingness to engage in similar behaviour.

As such, the aims of the current study were to (i) determine whether the MAKINGtheLINK intervention was associated with changes in stigmatizing attitudes over time; (ii) examine whether these changes prospectively predicted confidence to help a peer seek help and intentions to encourage help-seeking, and (iii) examine the relationship between changes in stigmatizing attitudes and participants’ use of alcohol (including the experience of alcohol-related problems) over the study period. No specific hypotheses regarding the latter relationship were generated as, to our knowledge, no studies have explored how changes in stigma influence alcohol use in young people.

## Methods

### Participants

Participants (n = 463) were a subset of a larger sample (n = 2447) participating in a randomized controlled trial of the MAKINGtheLINK intervention [19]. Twenty-two schools located within 50 km of metropolitan Melbourne, Australia, took part in the intervention, which was delivered to students in Year 9 (i.e., below the legal drinking age of 18). Schools were randomly allocated to the intervention and control groups according to the school’s Index of Socio-Educational Advantage (ICSEA) score, which has a mean of 1000 and standard deviation of 100, with two strata defined as < 1000 (‘disadvantaged’) and 1000 + (‘advantaged’) [20].

Students were assessed at baseline and three follow-ups that occurred 6-weeks, 6-months, and 12-months post-baseline, during which stigmatizing attitudes were assessed. However, due to concerns regarding the potential burden of the study on participants, questions regarding stigma were removed from the 6-month and 12-month surveys in some schools. Therefore, the 463 participants in the current study include only those who provided data on stigma at all four assessments; those who did not provide data at the later assessments (n = 1984) were excluded.

At baseline, there were no significant differences between the subset of 463 and the remainder of the sample in regards to age (F(1, 2408) = 1.627, *p *= 0.202) intentions to encourage help-seeking from peers (F(1, 2407) = 0.230, *p *= 0.631), family (F(1, 2406) = 2.648, *p *= 0.104), or the internet (F(1, 2407) = 0.004, *p *= 0.951), lifetime alcohol use (X^2^ = 1.726; *p *= 0.189) or past-year alcohol use (X^2^ = 2.94; *p *= 0.588). However, the sample included in the current study reported greater intentions to encourage help-seeking from formal sources (F(1,2406) = 14.71, *p *< 0.001), were more likely to have experienced one or more alcohol-related problem (X^2^ = 4.60, *p *= 0.032), and were more likely to be female (X^2^ = 155.30, *p *= <0.001).

## Intervention

Drawing upon two well-validated models of behaviour change, the Information-Motivation-Behavioural Skills Model (IMB) [21] and the Theory of Planned Behaviour (TpB) [22], the intervention consists of five interactive classroom activities run over two school periods, followed by a booster session 1 month later. The activities provide students with information about how and where to seek help, investigate psychological barriers to help-seeking, and examine risky behaviors associated with alcohol misuse and other mental health problems. According to our composite model, these activities (in conjunction with opportunities for skill rehearsal) will increase help-seeking intentions and behaviors. Specific activities covered include recognizing when a friend needs help, what types of helpers are available, myths and facts about substance use and mental health (including correcting misperceptions held by peers around substance use and mental health), identifying and overcoming barriers to professional help seeking, assisting a friend to access help, and accessing reliable sources of help.

## Measures

### Vignettes

Participants were presented with a vignette that described a young person (‘Samuel’) experiencing signs of alcohol misuse (Table 1). The description of ‘Samuel’ was adapted from a vignette depicting depression with comorbid alcohol misuse (based on DSM-IV alcohol abuse criteria), which has been used extensively in studies examining young people’s attitudes towards mental illness in Australia [16, 23–25]. Subsequent questions (including those relating to stigma, intentions to encourage help-seeking, and confidence to help a peer) were asked in relation to the problems described in the vignette.

**Table 1:** Alcohol misuse vignette

	Vignette
Alcohol misuse	Samuel is a close friend the same age as you. Lately, he’s been getting smashed nearly every weekend at parties and doing things that are really embarrassing. The other week he got drunk and vomited. Some girls that were at the party posted pictures of him on Facebook with his head over a toilet. He’s also been getting aggro when he drinks and people aren’t inviting your group to parties any more

### Stigma

Stigma was assessed using 6 items rated on a 5-point scale (1 = *strongly disagree* to 5 = *strongly agree*). Based on a previously published exploratory structural equation modeling analysis [26], two separate factors of stigma were calculated: ‘weak not sick’ (including the items “Sam could snap out of it if they wanted,” “Sam’s problem is a sign of personal weakness,” “Sam’s problem is not a real medical illness,” and “It is best to avoid Sam so that I don’t develop this problem”) and ‘dangerousness/unpredictability’ (including the items “Sam is dangerous” and “Sam’s problem makes them unpredictable”).

### Help-seeking

Intention to encourage a peer to seek help was measured via a modified version of the General Help Seeking Questionnaire (GSHQ; Wilson, 2005). This 15-item questionnaire required participants to indicate how likely they would be to encourage help-seeking for alcohol misuse from a range of sources each rated on a 5-point scale (1 = *very unlikely* to 5 = *very likely*). In line with previous studies [13] sources were divided into four categories: family (mother, father, other relative), peer (friend, boyfriend/girlfriend), internet (chat room/blog, website), and formal sources (school counsellor, mental health professional, alcohol and drug worker, GP).

### Confidence

Participants were asked how confident they would be to approach Samuel to talk about their concerns, rated on a 4-point scale from *1 *=* not confident* to *4 *=* very confident*.

### Alcohol use

Alcohol use was measured by adapting questions from the Australian Secondary School Students Alcohol and Drug (ASSAD) Survey [11]. Lifetime and past-year alcohol use were measured via the questions “how many times, if ever, have you drunk alcohol in your lifetime/over the past year.” There were seven fixed response options ranging from ‘never’ to ‘40 + times.’ Alcohol-related problems were assessed using questions from two large longitudinal studies, the Adolescent Temperament Project [27] and the International Youth Development Study [28]. Participants indicated whether or not they had experienced 10 alcohol-related problems over the past 6 months (e.g., get so drunk they were sick or passed out, have trouble at work, home, or school the next day, be unable to remember what happened the night before).

### Analysis

Repeated-measures ANOVA were used to determine whether the MAKINGtheLINK intervention was associated with changes in stigmatizing attitudes over time. Following this, a measure of overall change in stigmatizing attitudes was created by subtracting participants’ scores at baseline from their scores at the 12-month follow-up. A higher score on the change variable reflected an increase in stigma over time, while a lower score indicated a decrease in stigma. Separate linear regressions were used to examine whether change in stigma over time predicted (i) intentions and confidence to encourage a peer to seek help, (ii) past-year alcohol use, and (iii) past-year alcohol-related problems (all measured at the 12-month follow-up). Alcohol-related problems were assessed over a 6 month period, so data from the 6 and 12 month follow-ups were combined to create a past-year variable.

## Results

Baseline characteristics of the sample are presented in Table 2. There were significant differences between groups in ICSEA scores and gender, with the control group including a greater percentage of females and demonstrating higher ICSEA scores than the intervention group. Subsequent analyses controlled for these variables. There were no baseline differences between groups on any other variable.

**Table 2:** Baseline characteristics of control and intervention groups

Baseline characteristics	Control	Intervention	*p*^*a*^
Demographics			
ICSEA (M, SD)	1066.68 (43.46)	1033.09 (38.79)	< 0.001
Age (M, SD)	14.90 (0.41)	14.94 (0.40)	< 0.532
Age (range)	14.10–16.63	13.49–16.10	
Gender (female n, %)	150 (85.2%)	204 (71.0%)	< 0.001^b^
Stigmatising attitudes			
‘Weak not sick’ (M, SD)	2.74 (0.67)	2.76 (0.76)	0.729
’Dangerousness’ (M, SD)	3.65 (0.80)	3.68 (0.78)	0.631
Intentions			
Peer (M, SD)	3.08 (0.94)	3.13 (0.96)	0.57
Family (M, SD)	3.94 (0.93)	3.92 (1.01)	0.849
Formal (M, SD)	3.84 (0.68)	3.74 (0.77)	0.145
Internet (M, SD)	2.48 (1.00)	2.51 (1.03)	0.774
Confidence to help a peer (M, SD)	2.67 (0.88)	2.72 (0.83)	0.533
Alcohol use (n, %)	79 (45.6%)	114 (39.9%)	0.222^b^
Alcohol-related problems (n, %)	24 (30.3%)	27 (23.6%)	0.192^b^

^a^One-way ANOVA unless otherwise stated

^b^χ^2^

### Change in stigmatizing attitudes over time

There was no overall change in ‘weak-not-sick’ attitudes over the 12-month study period (F(3, 1368) = 1.678, *p *= <0.171), however there was a significant interaction between stigma and group (F(3, 1368) = 7.808, *p *= <0.001), with intervention participants reporting a greater reduction in stigma compared to controls. There was no association between change in stigma and gender (F(3, 1368) = 2.158, *p *= 0.093) or ICSEA scores (F(3, 1368) = 2.060, *p *= 0.105).

There was no overall change in perceptions of dangerousness over the 12-month study period (F(3, 1368) = 1.399, *p *= 0.242), and no difference in the rate of change between intervention and control participants (F(3, 1368) = 2.021, *p *= 0.110). There was no association between change in stigma and ICSEA scores (F(3, 1368) = 1410, *p *= 0.329), however there was a significant interaction between change in stigma and gender, with females reporting a greater decrease in stigma over time (F(3, 1368) = 4.623, *p *= 0.003).

### Change in stigmatizing attitudes and help-seeking confidence and intentions

The relationship between changes in stigmatizing attitudes and (i) participants’ intentions to encourage help-seeking and (ii) confidence to help a peer (both measured at the 12-month follow-up) are presented in Tables 3, 4. All analyses included group in addition to gender and ICSEA scores (the individual contribution of these variables is not reported here, however participation in the intervention significantly increased intentions to encourage help-seeking from formal sources and the internet, while females were less likely to encourage help-seeking from formal sources, for both sets of analyses). A decrease in ‘weak-not-sick’ attitudes between the baseline and 12-month follow-up assessments predicted stronger intentions to encourage a peer to seek help from family members and formal help sources at the 12-month follow-up assessment. A decrease in perceptions of dangerousness predicted greater confidence to help a peer at 12-months.

**Table 3:** Relationships between changes in ‘weak not sick’ attitudes over time, and intentions to encourage help-seeking and confidence to help a peer at 12-months

Intentions	Overall model^a^			‘Weak-not-sick’				
	R^2^	F(4455)	*p*	B	SE(B)	β	t	*p*
Peers	0.017	1.997	0.094	− 0.046	0.058	− 0.038	− 0.795	0.427
Family	0.030	3.492	0.008	− 0.147	0.055	− 0.127	− 2.699	0.005
Formal	0.065	7.946	< 0.000	− 0.091	0.041	− 0.102	− 2.21	0.028
Internet	0.028	3.298	0.011	0.069	0.064	0.050	1.068	0.286
Confidence	0.010	1.194	0.313	0.024	0.050	0.023	0.481	0.630

^a^Includes age, group, and ICSEA scores

**Table 4:** Relationships between changes in ‘dangerousness’ attitudes over time, and intentions to encourage help-seeking and confidence to help a peer at 12-months

Intentions	Overall model^a^			Dangerousness				
	R^2^	F(4455)	*p*	B	SE(B)	β	t	*p*
Peers	0.016	1.870	0.115	0.017	0.047	0.017	0.364	0.716
Family	0.015	1.686	0.152	0.018	0.045	0.019	0.401	0.689
Formal	0.059	7.097	< 0.001	0.044	0.034	0.059	1.294	0.196
Internet	0.031	3.681	0.006	− 0.085	0.053	− 0.075	− 1.622	0.105
Confidence	0.014	2.612	0.035	− 0.098	0.041	− 0.113	− 2.418	0.016

^a^Includes age, group, and ICSEA scores

### Associations between change in stigmatising attitudes and alcohol use

Lifetime use of alcohol increased from 42.0% of participants at baseline to 53.4% of participants at the 12-month follow-up. Amongst those who were non-drinkers at baseline, subsequent uptake of alcohol use was not associated with changes in ‘weak-not-sick’ attitudes (F(1, 253) = 0.299, *p *= 0.585) or perceptions of dangerousness (F(1, 253) = 0.153, *p *= 0.696). Amongst drinkers, past-year alcohol use and the number of alcohol-related problems experienced at the 12-month follow-up were both positively skewed, so logarithmic transformations were performed prior to analyses. Changes in ‘weak-not-sick’ attitudes were not associated with past-year alcohol use (r = 0.103, *p *= 0.104) or the number of alcohol-related problems experienced over this period (r = 0.024, *p *= 0.820). Similarly, changes in perceptions of dangerousness were not associated with past-year alcohol use (r = 0.056, *p *= 0.382) or the number of alcohol-related problems experienced (r = 0.121, *p *= 0.260). There were no differences between intervention and control groups in regard to the uptake of alcohol use (χ^2^ = 1403, *p *= 0.236), past-year use (F(1249) = 0.007, *p *= 0.933), or the experience of alcohol-related problems (F(1, 87) = 0.488, *p *= 0.487).

## Discussion

These findings provide partial support for the hypotheses. Participation in the MAKINGtheLINK intervention was associated with a greater reduction in ‘weak-not-sick’ attitudes at the 12-month follow-up, and decreases in this dimension of stigma predicted stronger intentions to encourage help-seeking from family members and formal help sources at the 12-month follow-up. However, the intervention was not associated with changes in intentions to encourage help-seeking from peers or the internet. Conversely, perceptions of dangerousness did not differ between intervention and control participants, and did not change significantly over the four assessment points. Decreases in this dimension of stigma predicted increased confidence to help a peer at the 12-month follow-up, but were not associated with intentions to encourage help-seeking from any source.

Previously, we found that greater ‘weak-not-sick’ attitudes were associated with weaker intentions to seek help from peers, family, formal sources, and the internet [15]. The present study provides evidence that reducing this dimension of stigma increases adolescents’ likelihood to encourage help-seeking from family members and formal help sources. These two sources are arguably of most importance in ensuring that young people get help when needed: seeking formal help early is crucial in ameliorating the long-term effects of substance use disorders [29], and families can play a central role in facilitating this process during adolescence [30]. Moreover, family members are more likely to recommend seeking appropriate treatment (e.g., seeing a GP) than other sources of help, such as peers [30, 31]. Is unclear why the effect of reducing weak-not-sick attitudes did apply to peers, however it is possible that adolescents’ motives to encourage help-seeking from their own social groups change over time; as noted by Adlof and colleagues, stigma is lower amongst adolescents with substance-using social groups. As such, encouraging help-seeking from other young people may become more strongly linked to social factors (e.g., group norms) than individual attitudes towards drinking, as alcohol use becomes more prevalent as they age.

More targeted interventions may be necessary to reduce perceptions of dangerousness. These could emphasize biopsychosocial explanations of mental illness, which appear to reduce stereotypes of dangerousness and social distance specifically, or could incorporate contact in addition to education, as this may have a broader effect on stigmatising attitudes [32]. However, it will remain important to evaluate the impact of these young people’s willingness to engage with and help their peers. Although perceptions of dangerousness were not associated with helping intentions in the current study, the relationship of these variables at baseline suggest that reducing this dimension of stigma may *decrease* intentions to encourage help-seeking from formal help sources, which could result in a reduction in opportunities for at-risk adolescents to access professional treatment. Conversely, perceptions of dangerousness have been found to increase negative interpersonal outcomes [1], and reducing these attitudes would therefore have social benefits for young people who might be avoided or feared due to their alcohol use [6]. Ultimately, further research is needed to understand how perceptions of dangerousness affect how adolescents respond to alcohol problems in their peers.

While knowledge regarding the relationship between stigma and substance use in young people is limited [33], it has been suggested that reducing the stigma associated with alcohol misuse may result in harmful drinking [18]. The results of the current study were not consistent with this hypothesis, as changes in ‘weak not sick’ attitudes and perceptions of dangerousness were not associated with the initiation of alcohol use, or the frequency of drinking or experience of alcohol-related problems at the 12-month follow-up. This finding is of particular interest given the significant decrease in ‘weak not sick’ attitudes evident across the whole sample: being less likely to see alcohol misuse as a sign of weakness in others may pose little risk in regard to normalising problematic drinking behaviour. However, it should be noted that due to the design of the study, changes in stigmatising attitudes and alcohol use and problems were measured over the same 12-month time-frame. Future studies examining this issue may wish to utilise a prospective design in which changes in stigmatising attitudes are measured prior to assessing drinking behaviour. Examining drinking behaviour at a later age, where there is likely to be more variation in recent consumption and associated problems, is also necessary, as approximately half of the current sample were non-drinkers at all time points.

There are a number of limitations to note. First, only 463 of the full sample of 2447 participants completed measures of stigma at at all time points. A loss of statistical power may explain the relative lack of significant relationships between changes in stigma and helping intentions, compared to the results of previous cross-sectional analyses at baseline [15]. This may also have led to biases in the current results, particularly in regard to formal help-seeking intentions, as these were found to be significantly higher at baseline among the sample included in the current study. Second, we assessed intentions, rather than actual helping behaviour. While intentions measured by the GHSQ have been found to reliably predict both past and future behaviour [34], analysis of baseline data from the same sample found that dangerousness was associated with increased intentions to encourage formal help-seeking, but also with decreased rates of actual helping behaviour in the past [15]. Third, the gender of the peer described in the vignette was fixed (i.e., all vignettes described a male named ‘Samuel’), which, based on previous research, could have influenced adolescents’ responses [17]. However, gender was included in all analyses, and demonstrated few associations with the variables of interest. In addition, the same analyses run without including gender (not reported here) did not affect the significance of the overall findings. The interpersonal conflict and social exclusion described in the vignette may also have influenced participants’ help-seeking preferences, as if a peer is behaving aggressively and no longer being invited to social events, help-sources such as family members or health professionals may be perceived as more appropriate or effective sources of help. Future studies may wish to develop vignettes depicting varied negative consequences of alcohol abuse to determine whether there is a relationship between problem type and preference for a particular help source. Finally, the intervention was trialled in a community-based sample where less than 3% of participants had ever sought help for alcohol or other drug problems at baseline [13], and results therefore may not generalise to higher-risk groups.

## Conclusions

This study examined the effect of a school-based help-seeking intervention on stigmatising attitudes towards alcohol misuse, as well as the relationship between reductions in stigma and intentions to encourage help-seeking among their peers. Results indicate that the MAKINGtheLINK intervention is effective in reducing ‘weak-not-sick’ attitudes towards alcohol misuse during adolescence, which in turn appear to increase intentions to encourage help-seeking from family members. In contrast, the intervention was not as successful in reducing perceptions of dangerousness over time. Overall, the findings demonstrate the complex relationships that exist between stigma, intentions, and behaviours, and highlight the importance of gaining a better understanding of how these factors relate to alcohol misuse during adolescence.

## Data Availability

Due to ethics agreements associated with the trial, participant data is not available to external sources.
